# Supplementary material for: Integrated multimodality microscope for accurate and efficient target-guided cryo-lamellae preparation
Source: Nat Methods. 2023 Jan 16;20(2):268–75. doi: 10.1038/s41592-022-01749-z (PMC9911353; doi:10.1038/s41592-022-01749-z)
Supplement: Supplementary file 1 — Supplementary Figs. 1–7, Notes 1 and 2 and Tables 1 and 2. [file 41592_2022_1749_MOESM1_ESM.pdf]

nature methods

Article https://doi.org/10.1038/s41592-022-01749-z

Integrated multimodality microscope for accurate and efficient target-guided cryo-lamellae preparation

In the format provided by the authors and unedited

Supplementary Information

**Supplementary Figure 1**	Architecture and setup of the CLIEM system
**Supplementary Figure 2**	Optical and mechanical design of the integrated confocal microscope
**Supplementary Figure 3**	Optical performance of the integrated confocal microscope
**Supplementary Figure 4**	Custom-built cryogenic sample loading module based on the Gatan 910 cryo-holder
**Supplementary Figure 5**	Determination of the TOI position to the prelamella boundaries
**Supplementary Figure 6**	Fluorescence images of the LD-mitochondria reporter
**Supplementary Figure 7**	Fluorescence images of the MERC reporter
**Supplementary Note 1**	Optical performance of the integrated confocal microscope
**Supplementary Note 2**	Cryogenic sample loading module and workflow
**Supplementary Table 1**	Parameters of cryo-ET data collation
**Supplementary Table 2**	Success rate of targeted-FIB milling of biological samples

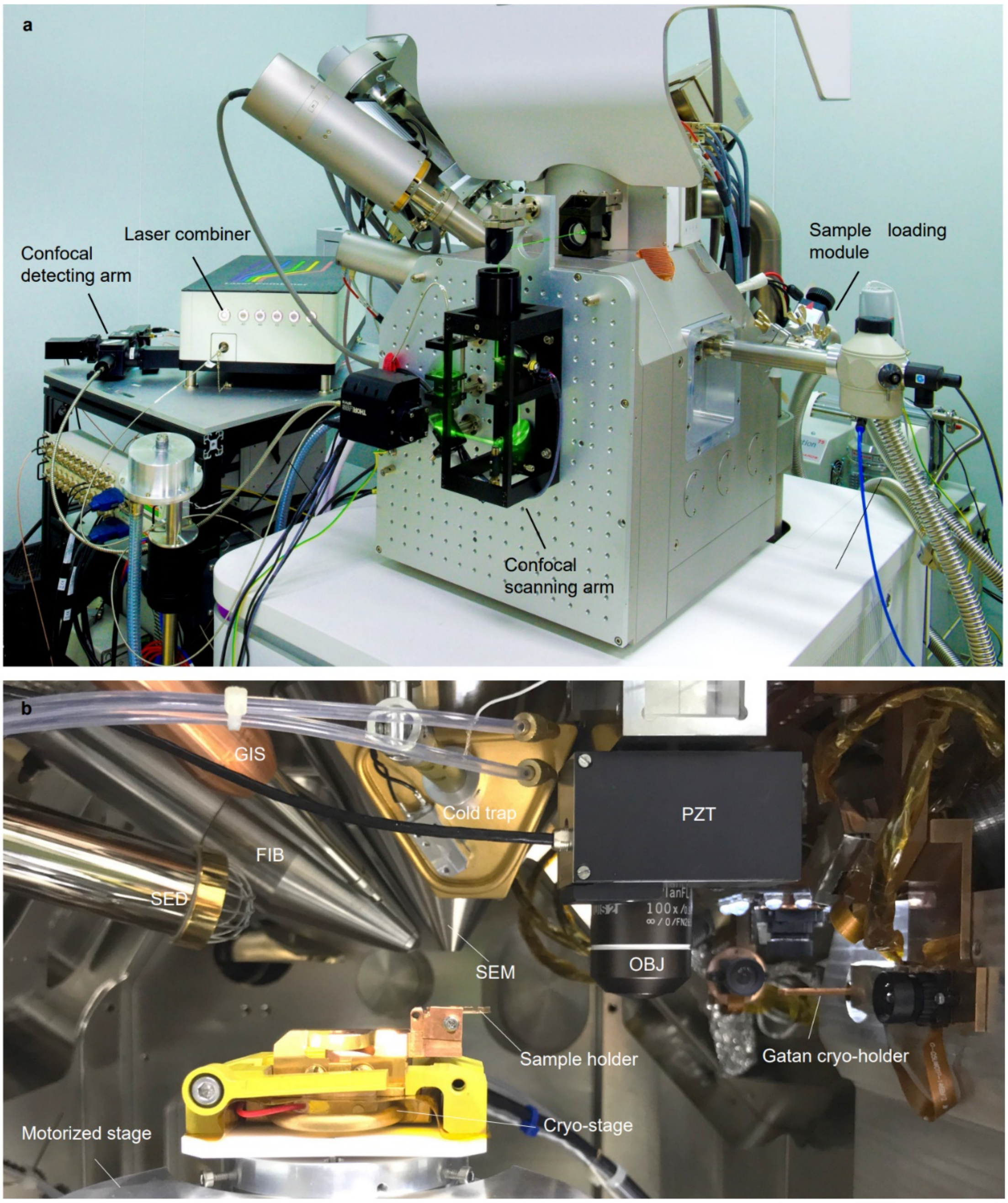

Supplementary Figure 1. Architecture and setup of the CLIEM system.

a, A photograph of the exterior of the CLIEM system. **b,** A photograph of the interior of the CLIEM system.

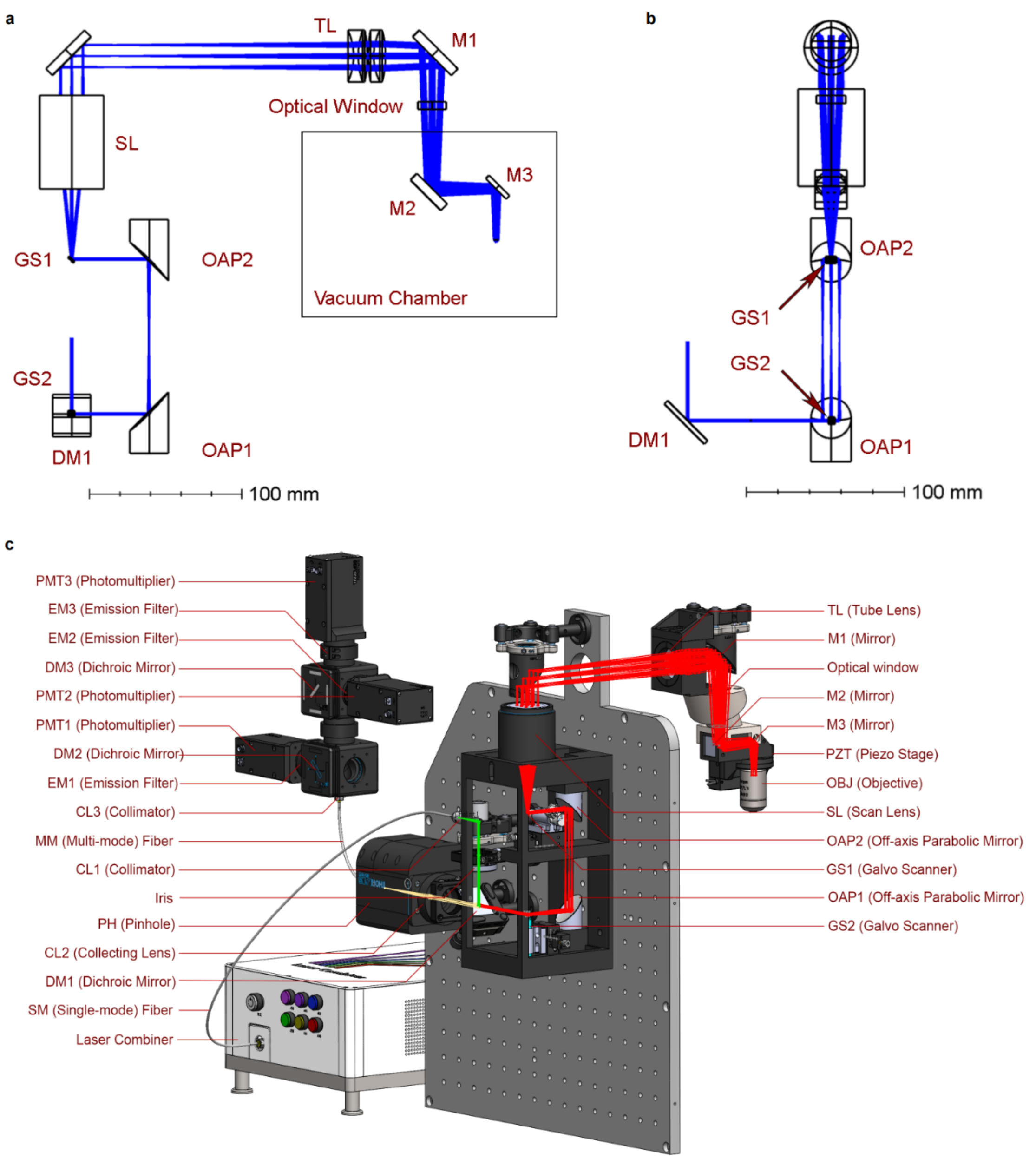

Supplementary Figure 2. Optical and mechanical design of the integrated confocal microscope.

a, Side view of the optical path design of the confocal microscope. **b,** Top view of the optical path design of the confocal microscope. **c,** 3D rendering of the integrated confocal microscope. 405 nm Laser (WCP405-150FS-224, Pavilion Integration Corp.), 488 nm Laser (WCP488-150FS-224, Pavilion Integration Corp.), 532 nm Laser (WCP532-100FS-224, Pavilion Integration Corp.), 561 nm Laser (LS 150 mW, OBIS), 638 nm Laser (WCP405-150FS-224, Pavilion Integration Corp.), Fiber Coupler (HPUC-23AF-400/700-P-4.5AC-15, OZ Optics), SM Fiber (QPMJ-3AF3S-400-3/125-3AS-3-1, OZ Optics), Collimator (RC08FC-P01, Thorlabs Inc.), Iris (SM1D12SZ, Thorlabs Inc.), DM1 (ZT405/488/561/647-rpc, Chroma), GS1, GS2 (6215H, Cambridge Technology), OAP1,OAP2 (MPD129-P01, Thorlabs Inc.), SL (CLS-SL, Thorlabs Inc.), TL (SWTLU-S, Olympus), Optical Window

(10Q20RAR.L, Newport), PZT (nPFocus250HV, nPoint Inc.), OBJ (MPLFLN 100× NA0.9, Olympus), PH (MPH16, Thorlabs Inc.), MM fiber (customized, NA0.22, core diameter 1000 μm), DM2 (T550lpxr, Chroma), DM3 (T635lpxr, Chroma), EM1 (ZET405/488m, Chroma), EM2 (ZET488/561m, Chroma), EM3 (ZET405/488/561/640m or ZET405/488/561, Chroma), PMT1, PMT2, PMT3 (PMT2101/M, Thorlabs Inc.).

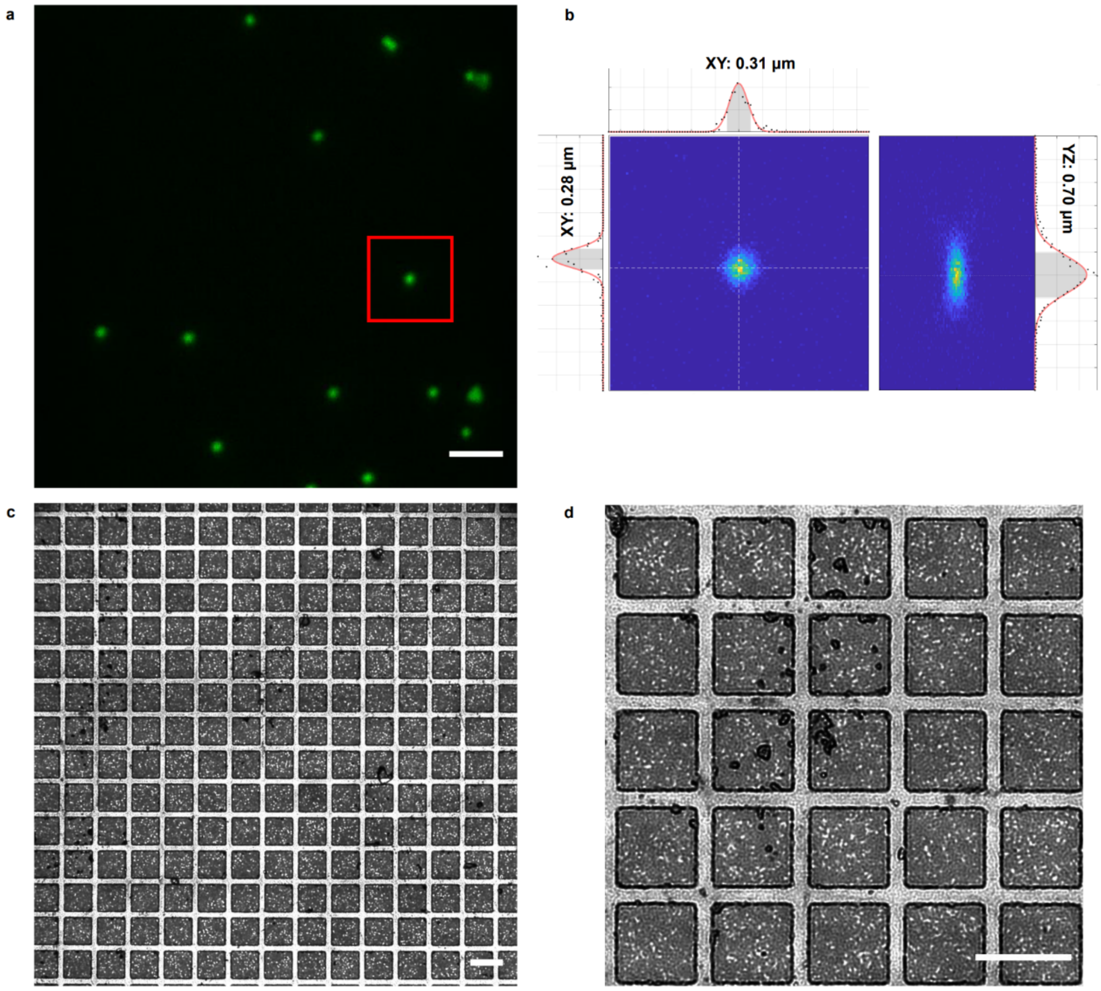

Supplementary Figure 3. Optical performance of the integrated confocal microscope.

a, Fluorescence image of 0.2 µm fluorescent beads (T7280, TetraSpeck, Thermo Fisher Scientific) for resolution calibration using Olympus MPLFLN 100× NA 0.9 objective. A 488 nm Laser was used for excitation and PMT1 was used for detection. The X, Y, and Z pixel sizes were 34.7 nm, 34.7 nm, and 50 nm, respectively. One bead (boxed) was chosen for resolution calibration. Scale bar, 2 µm. **b,** 3D resolution determination of the integrated confocal microscope. PSF was analyzed in Matlab 2017b using opensource code (Rob Campbell, measurePSF, https://github.com/raacampbell/measurePSF, GitHub). The full width half maximum (FWHM) of Gaussian fitted curves along X-Y, Y-X, and Y-Z directions were 0.31 µm, 0.28 µm, and 0.70 µm, resulting in the lateral and axial resolutions of 295 nm and 700 nm, respectively. Ten experiments were repeated independently with similar results. **c,d**, Confocal BF images of a calibration target (Thorlabs, R1L3S5P) for FOV and pixel size calibration, showing an FOV of 145 µm x 145 µm in **c** and 50 µm x 50 µm in **d**. Scale bar in **c, d**, 10 µm.

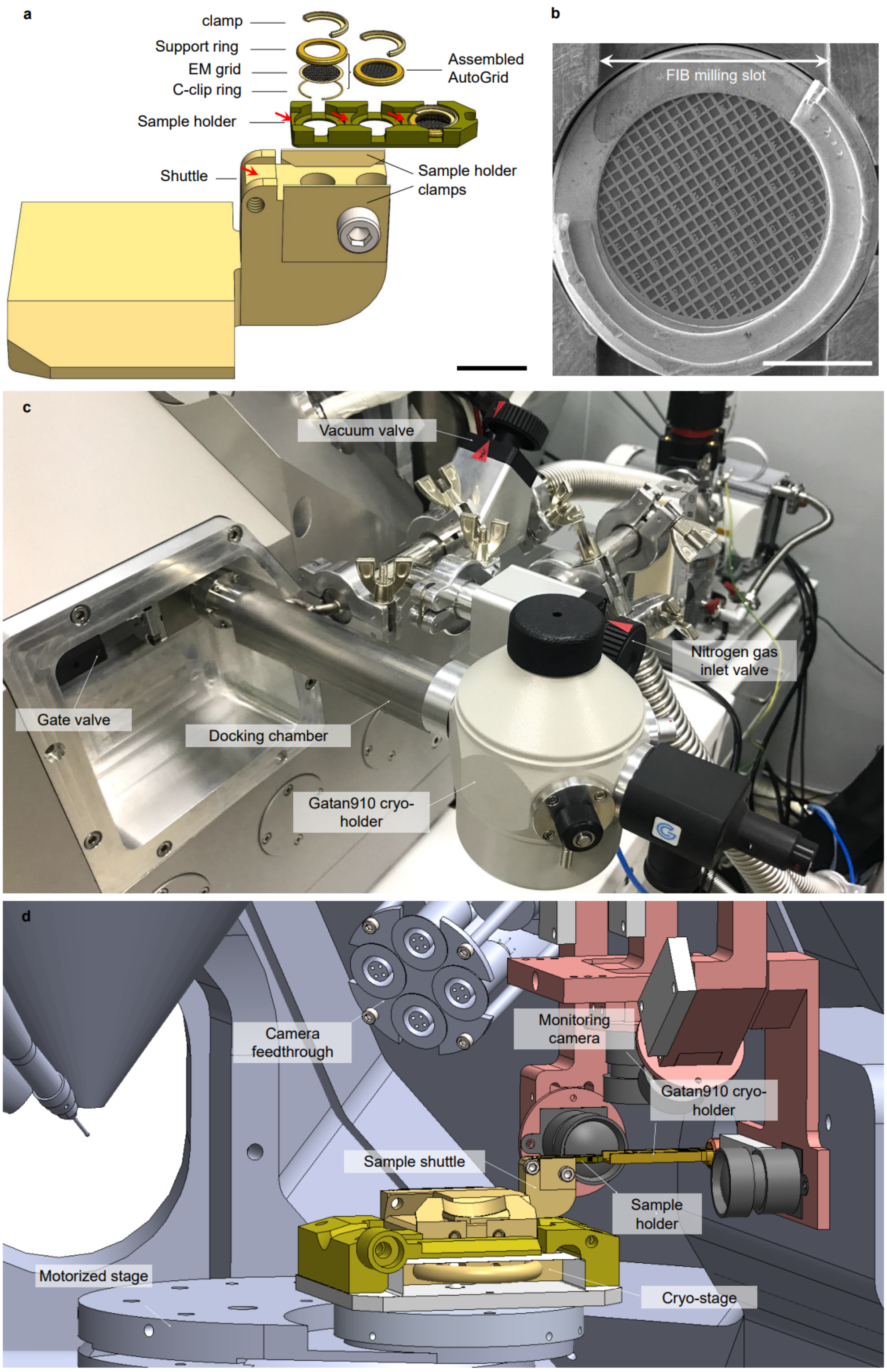

Supplementary Figure 4. Custom-built cryogenic sample loading module based on the Gatan 910 cryo-holder.

a, 3D rendering of the custom-designed sample holder, which accommodated three AutoGrid samples. Slots (arrows) were made on the sample holder and the shuttle to let FIB through during milling. Scale bar 5 µm. **b,** An SEM micrograph of an AutoGrid mounted in the sample holder,

displaying the FIB milling slot. Scale bar 1 mm. **c,** A photograph of the sample loading module outside the vacuum chamber. **d,** 3D rendering of the sample loading module inside the vacuum chamber.

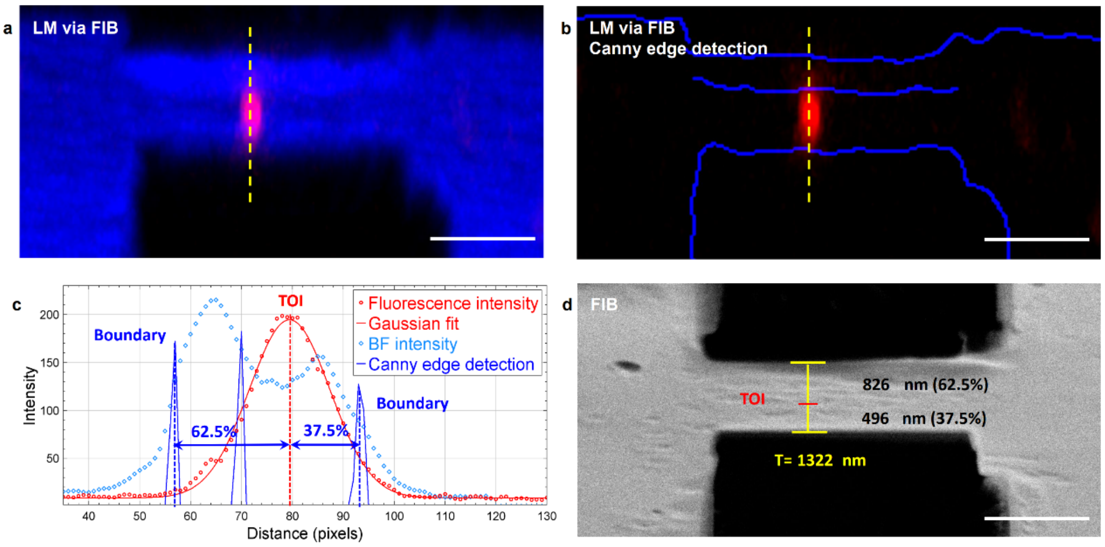

Supplementary Figure 5. Determination of the TOI position to the prelamella boundaries.

a, LM via FIB of the prelamella. To obtain the LM via FIB image, raw 3D images were first tilted 72 degrees using Fiji (Volume Viewer plugin) in Max Projection display mode. Interpolation was performed using the Tricubic sharp algorithm in Volume Viewer plugin with a Z-aspect of 4.046. The yellow dashed line indicates the position for intensity profile analysis in **c**. **b,** LM via FIB after Canny edge detection on BF image. Edge detection was performed on the BF image using Canny Edge Detector plugin of Fiji with the following parameters. Gaussian kernel radius 3.5, Low threshold 2.5, and High threshold 7.0. The BF image after edge detection was merged with fluorescence image. The yellow dashed line indicates the position for intensity profile analysis in **c**. **c,** Line profile across the TOI in **a** and **b**. The red circles represent the intensity of the fluorescence image, and the red line represents the Gaussian fitted curve to the fluorescence profile. The blue diamonds represent the intensity of the BF image, and the blue lines show the profile of the BF image after edge detection. TOI's position was determined from the peak of the fitted curve as 79.5, and the positions of the boundaries were determined from the peaks of the edge detection as 57 and 93, respectively. The relative position of the TOI with respect to the boundaries was measured in percentage as 62.5% and 37.5%, respectively. **d,** FIB image of the prelamella. The thickness (T) of the pre-lamella was measured with nanometer precision in the FIB-SEM control software, and the TOI position was determined according to the measured relative position in **c**. The thickness of the prelamella measured in LM via FIB was 1780 nm, while 1322 nm in the FIB

image. The difference results from the fitting error in LM via FIB image and manual measurement error in FIB image. Therefore, the relative position of the TOI to the boundaries in percentage was used as the guidance to determine the TOI position in the FIB image. Scale bar, 2 μm

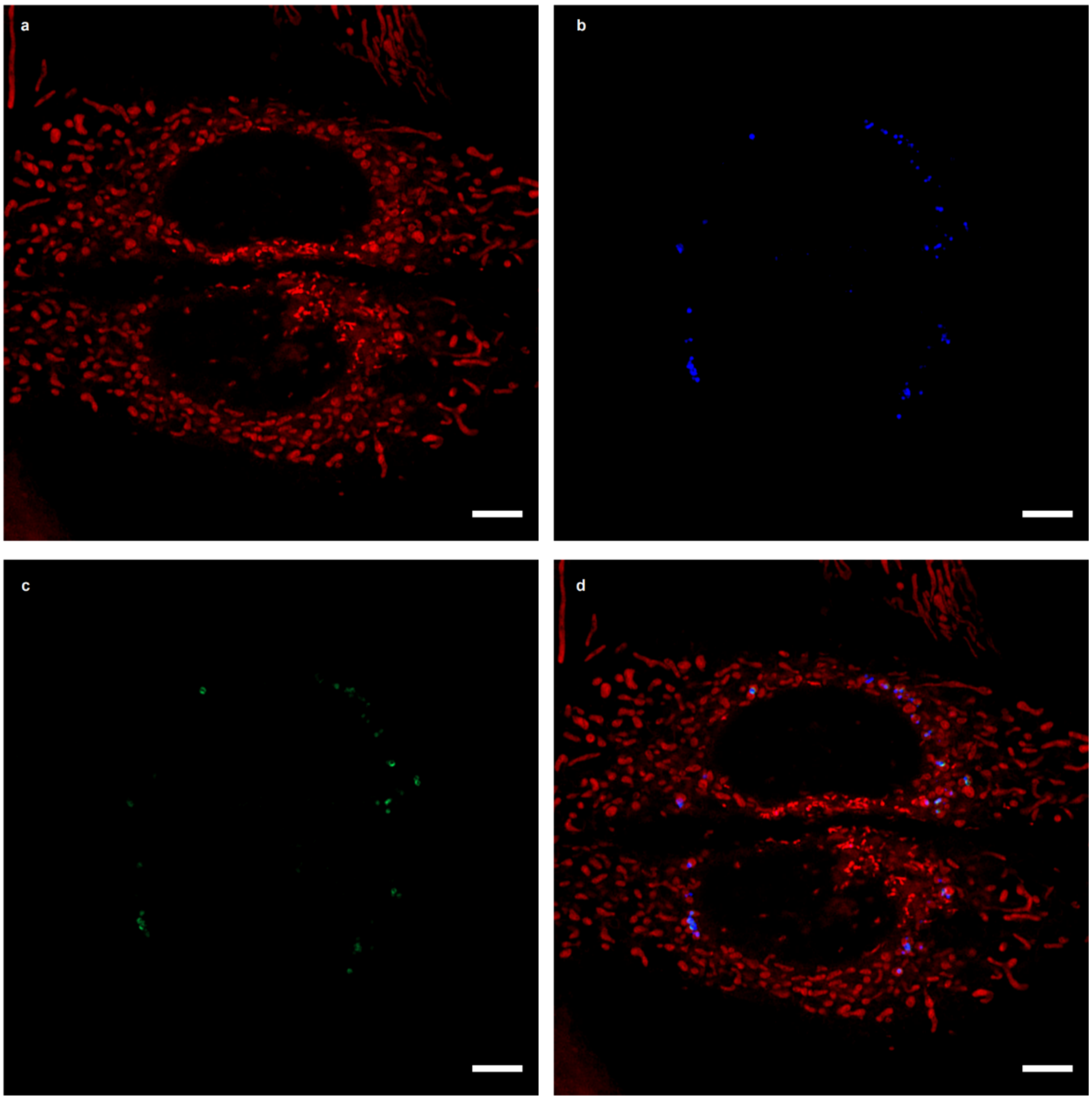

Supplementary Figure 6: Fluorescence images of the LD-mitochondria reporter.

The HepG2 cells with LD-mitochondria reporter were incubated with MitoTracker and LipidTox to stain mitochondria and lipid droplets, respectively. The fluorescence images were acquired with a Zeiss LSM980 microscope equipped with a 63x / NA1.40 oil immersion objective (Zeiss). **a,** MitoTracker. **b,** LipidTox. **c,** GFP. **d,** Merge. Scale bar, 5 µm. Five experiments were repeated independently with similar results.

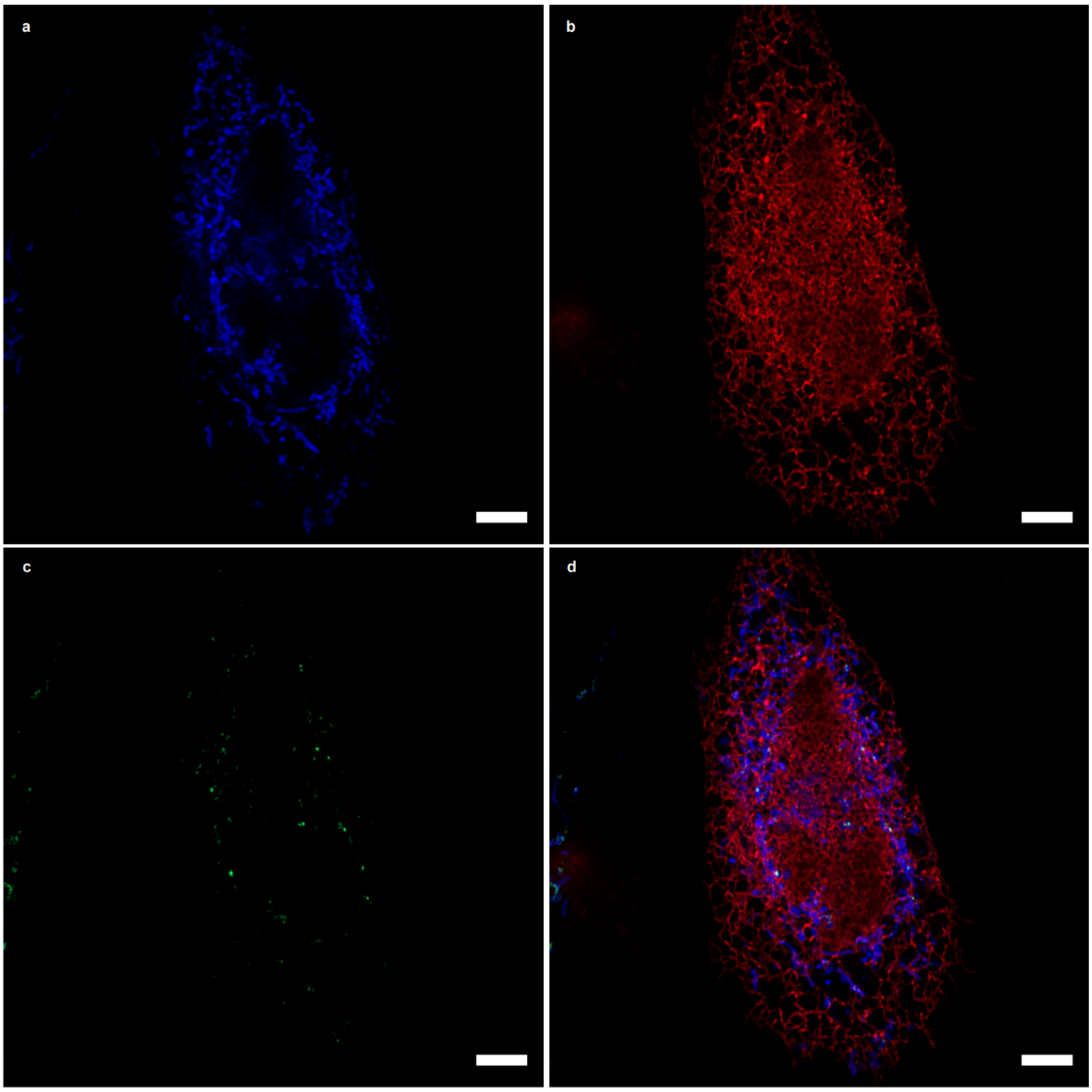

Supplementary Figure 7. Fluorescence images of the MERC reporter.

The HepG2 cells with MERC reporter were transiently transfected with ER-DsRed for ER labeling and then incubated with MitoTracker to stain mitochondria. The fluorescence images were acquired with the Zeiss LSM980 microscope equipped with a 63x/1.40 NA oil immersion objective (Zeiss). **a**, MitoTracker. **b**, ER-DsRed. **c**, GFP. **d**, Merge. Scale bar, 5 µm. Five experiments were repeated independently with similar results.

Supplementary Note 1: Optical performance of the integrated confocal microscope.

Optical resolution evaluation. To calibrate the resolution of the optical system, fluorescent beads with a diameter of 0.2 µm were diluted and spread on a 400 mesh copper EM grid (BZ11024a, Beijing Zhongjingkeyi Technology Co., Ltd.). Then the beads were imaged under cryogenic conditions with a dry objective (MPLFLN 100× NA 0.9, Olympus) using a 488 nm excitation wavelength, a 25 µm pinhole size, and X, Y, and Z pixel sizes of 34.7 nm, 34.7 nm, and 50 nm, respectively (Supplementary Fig. 3a). Gaussian fitting was performed in the X-Y, Y-X, and Y-Z directions, and the full width half maximum (FWHM) was calculated. The lateral resolution was measured as the averaged FWHM of X-Y and Y-X to be 295 nm, whereas the axial resolution was measured as the FWHM of Y-Z to be 700 nm (Supplementary Fig. 3b).

FOV and pixel size calibration. The optical window that is mounted on the vacuum flange limited the system's nonvignetted field of view to 145 µm x 145 µm when using an Olympus 100X objective (Supplementary Fig. 3c). The physical size of FOV was calibrated using a standard target (Thorlabs, R1L3S5P). The flexibility of FOV adjustment is shown by acquiring a small FOV of 50 µm x 50 µm (Supplementary Fig. 3d). The pixel size was calibrated by measuring the distance in pixels and in real sizes based on the image of the standard target.

Supplementary Note 2: Cryogenic sample loading module and workflow.

We have designed a special sample holder (Supplementary Fig. 4a) to load three standard AutoGrids onto the cryostage. The AutoGrids were mounted in the sample holder with the support ring facing up, and were fixed by clamps. The distance from the upper surface of the sample holder to the surface of the EM grid is approximately 0.5 mm, which was within the WD of the objective used (NA0.9, WD 1 mm). The sample holder could slide onto the shuttle and be fixed in position by two clamps. The sample holder and the shuttle also had FIB milling slots that provide space for the FIB during milling (Supplementary Fig. 4b). Furthermore, we designed a docking module for sample loading without breaking the vacuum of the system.

During sample loading, the Gatan 910 holder was first inserted into the docking chamber (Supplementary Fig. 4c), and vacuum was generated in the chamber. The gate valve was then opened, and the cryo-holder was inserted into the vacuum chamber. Afterward, the sample holder could be loaded/unloaded onto/from the sample shuttle by moving the motorized stage (Supplementary Video 2). Three cameras were used to monitor the manual movement of the cryo-stage (Supplementary Fig. 4d). When removing the cryo-holder from the vacuum chamber, the reverse process was performed, and dry Nitrogen gas was fed into the docking chamber to eliminate the vacuum and prevent ice formation on the sample.

Supplementary Table 1. Parameters of cryo-ET data collection

	LD-mitochondria	MERC	Centrosome
Microscope	Titan Kris G2	Titan Krios G2	Titan Krios G2
Operation voltage (kV)	300	300	300
Detector	Gatan K2	Gatan K2	Gatan K2
Mode	Counting	Counting	Counting
Frame rate (fps)	10	16	10
Magnification	26,000 x	26,000 x	33,000 x
Pixel size (Å)	5.42	5.42	4.3
Exposure time (s)	4.5	4.8	3
Tilting angle (°)	-54 - 48	-40 - 60	-40 - 36
Angle increment (°)	3	2.5	2
Dose per tilt (e/Å^2)	105	123	117
Defocus (μm)	-6	-6	-5
Direction	Unidirectionally	Unidirectionally	Unidirectionally

Supplementary Table 2. Success rate of targeted-FIB milling of biological samples

	LD-mitochondria	MERC	Centrosome	Overall
Number of prepared lamellae	31	5	6	42
Number of prepared lamellae containing desired feature	29	5	6	40
Success rate	94%	100%	100%	95%
