# Supplementary material for: Integrated multimodality microscope for accurate and efficient target-guided cryo-lamellae preparation
Source: Nat Methods. 2023 Jan 16;20(2):268–75. doi: 10.1038/s41592-022-01749-z (PMC9911353; doi:10.1038/s41592-022-01749-z)
Supplement: Supplementary file 2 — Reporting Summary [file 41592_2022_1749_MOESM2_ESM.pdf]

nature research

Corresponding author(s): Qiang Guo, Tao Xu, Wei Ji

Last updated by author(s): Nov 6, 2022

Reporting Summary

Nature Research wishes to improve the reproducibility of the work that we publish. This form provides structure for consistency and transparency in reporting. For further information on Nature Research policies, see our Editorial Policies and the Editorial Policy Checklist.

Statistics

For all statistical analyses, confirm that the following items are present in the figure legend, table legend, main text, or Methods section.

n/a	Confirmed	
☐	☒	The exact sample size (*n*) for each experimental group/condition, given as a discrete number and unit of measurement
☒	☐	A statement on whether measurements were taken from distinct samples or whether the same sample was measured repeatedly
☒	☐	The statistical test(s) used AND whether they are one- or two-sided<br>*Only common tests should be described solely by name; describe more complex techniques in the Methods section.*
☒	☐	A description of all covariates tested
☒	☐	A description of any assumptions or corrections, such as tests of normality and adjustment for multiple comparisons
☒	☐	A full description of the statistical parameters including central tendency (e.g. means) or other basic estimates (e.g. regression coefficient) AND variation (e.g. standard deviation) or associated estimates of uncertainty (e.g. confidence intervals)
☒	☐	For null hypothesis testing, the test statistic (e.g. F, t, r) with confidence intervals, effect sizes, degrees of freedom and P value noted<br>*Give P values as exact values whenever suitable.*
☒	☐	For Bayesian analysis, information on the choice of priors and Markov chain Monte Carlo settings
☒	☐	For hierarchical and complex designs, identification of the appropriate level for tests and full reporting of outcomes
☒	☐	Estimates of effect sizes (e.g. Cohen's *d*, Pearson's *r*), indicating how they were calculated

Our web collection on statistics for biologists contains articles on many of the points above.

Software and code

Policy information about availability of computer code

Data collection	TESCAN Essence 1.1.4.0 for system control and FIB/SEM image acquisition, C++ 11 with custom code for system control and LM image acquisition, SerialEM 3.8 for cryo-ET data collection.
Data analysis	SolidWorks 2019 for mechanical design, Zemax OpticStudio 16.5 SP5 for optical design, Fiji 1.53f51 for image analysis, measurePSF code in MATLAB R2017b for PSF analysis, Huygens 20.04 for LM image deconvolution, IMOD 4.11.0 for tomographic reconstruction, RELION v2.1 and Dynamo 1.1.532 for subtomogram averaging, Imaris 9.8.0 for segmentation and visualization, ChimeraX 1.3 for 3D rendering.

For manuscripts utilizing custom algorithms or software that are central to the research but not yet described in published literature, software must be made available to editors and reviewers. We strongly encourage code deposition in a community repository (e.g. GitHub). See the Nature Research guidelines for submitting code & software for further information.

Data

Policy information about availability of data

All manuscripts must include a data availability statement. This statement should provide the following information, where applicable:

- Accession codes, unique identifiers, or web links for publicly available datasets
- A list of figures that have associated raw data
- A description of any restrictions on data availability

The tomographic reconstructions in this work have been deposited in the Electron Microscopy Database (EMDB) with the accession codes EMD-33496 (LD-mitochondria), EMD-33495 (centrosome). Other source data that support the findings of this study are available from the corresponding author upon request.

Field-specific reporting

Please select the one below that is the best fit for your research. If you are not sure, read the appropriate sections before making your selection.

☒ Life sciences ☐ Behavioural & social sciences ☐ Ecological, evolutionary & environmental sciences

For a reference copy of the document with all sections, see nature.com/documents/nr-reporting-summary-flat.pdf

Life sciences study design

All studies must disclose on these points even when the disclosure is negative.

Sample size	The number of lamellae prepared for LD-mitochondria, MERC and centrosome experiments is n=31, n=5, n=6, respectively. The number of cryo-ET tilt series collected for LD-mitochondria, MERC and centrosome experiments is n=5, n=3, n=3, respectively. We determined these sample sizes to be sufficient because the targets of interests could be resolved with desired resolution using cryo-ET.
Data exclusions	No data was excluded from the study.
Replication	For LM-guided FIB-milling, the number of final lamellae that contained the desired structures after FIB-milling is n=29, n=5, n=6 for LD-mitochondria, MERC and centrosome experiments, respectively, resulting in an overall 95% replication rate. For cryo-ET experiments, all collected cryo-ET tilt series, in total n=5, n=3, n=3 for LD-mitochondria, MERC and centrosome experiments, respectively, delivered desired target structures.
Randomization	Cells were randomly selected for lamellae preparation. Lamellae that contained minimal ice contamination and breakage were selected for tilt series data collection.
Blinding	Blinding is considered not necessary for the method, because the purpose of this work was to demonstrate a new technique, and this technique does not depend on the statistical variation of the properties of the samples. For the cell experiments, blinding was not possible because the experimental conditions were evident from the image data.

Reporting for specific materials, systems and methods

We require information from authors about some types of materials, experimental systems and methods used in many studies. Here, indicate whether each material, system or method listed is relevant to your study. If you are not sure if a list item applies to your research, read the appropriate section before selecting a response.

Materials & experimental systems

n/a	Involved in the study	
☒	☐	Antibodies
☐	☒	Eukaryotic cell lines
☒	☐	Palaeontology and archaeology
☒	☐	Animals and other organisms
☒	☐	Human research participants
☒	☐	Clinical data
☒	☐	Dual use research of concern

Methods

n/a	Involved in the study	
☒	☐	ChIP-seq
☒	☐	Flow cytometry
☒	☐	MRI-based neuroimaging

Eukaryotic cell lines

Policy information about cell lines

Cell line source(s)	HepG2 cell line (1101HUM-PUMC000035, National Infrastructure of Cell Line Resource, China). Hela cell line (ATCC, CCL-2) was provided from Prof. Jianguo Chen's lab at Peking University.
Authentication	The cell lines were not further authenticated in our lab.
Mycoplasma contamination	All cell lines used in this study tested negative for mycoplasma contamination.
Commonly misidentified lines (See ICLAC register)	No commonly misidentified lines were used in the study.
